# mm-TPG: Traffic Policemen Gesture Recognition Based on Millimeter Wave Radar Point Cloud

**DOI:** 10.3390/s23156816

**Published:** 2023-07-31

**Authors:** Xiaochao Dang, Wenze Ke, Zhanjun Hao, Peng Jin, Han Deng, Ying Sheng

**Affiliations:** 1 College of Computer Science and Engineering, Northwest Normal University, Lanzhou 730070, China; 2021222210@nwnu.edu.cn (W.K.); haozhj@nwnu.edu.cn (Z.H.); 2021222246@nwnu.edu.cn (P.J.); qfsfdenghan@163.com (H.D.); 2 Gansu Province Internet of Things Engineering Research Center, Lanzhou 730070, China; 3 School of Physics and Electrical Engineering, Northwest Normal University, Lanzhou 730070, China; 13210828678@163.com

**Keywords:** automatic driving, gesture recognition, millimeter-wave radar, point cloud

## Abstract

Automatic driving technology refers to equipment such as vehicle-mounted sensors and computers that are used to navigate and control vehicles autonomously by acquiring external environmental information. To achieve automatic driving, vehicles must be able to perceive the surrounding environment and recognize and understand traffic signs, traffic signals, pedestrians, and other traffic participants, as well as accurately plan and control their path. Recognition of traffic signs and signals is an essential part of automatic driving technology, and gesture recognition is a crucial aspect of traffic-signal recognition. This article introduces mm-TPG, a traffic-police gesture recognition system based on a millimeter-wave point cloud. The system uses a 60 GHz frequency-modulated continuous-wave (FMCW) millimeter-wave radar as a sensor to achieve high-precision recognition of traffic-police gestures. Initially, a double-threshold filtering algorithm is used to denoise the millimeter-wave raw data, followed by multi-frame synthesis processing of the generated point cloud data and feature extraction using a ResNet18 network. Finally, gated recurrent units are used for classification to enable the recognition of different traffic-police gestures. Experimental results demonstrate that the mm-TPG system has high accuracy and robustness and can effectively recognize traffic-police gestures in complex environments such as varying lighting and weather conditions, providing strong support for traffic safety.

## 1. Introduction

With the continuous development of the economy, people’s living standards are improving and their requirements for travel are also rising. The number of motor vehicles in cities is growing rapidly, yet the carrying capacity of roads is limited. During peak holiday periods or bad weather conditions, the number of vehicles on the road can increase dramatically, leading to serious traffic congestion. Traditional traffic facilities such as fixed traffic signs and electronic traffic signals are no longer able to relieve congestion quickly and effectively so traffic management authorities usually dispatch experienced traffic-police officers to congested intersections to direct and divert traffic in order to restore normal traffic as soon as possible. However, with the rapid development of autonomous driving technology, traffic-police gesture recognition technology has become the key to solving road congestion and traffic management problems. Traffic-police gesture recognition technology is widely used in the field of traffic management and traffic safety but it can also be used in the field of intelligent transportation systems and autonomous driving to improve traffic management efficiency and reduce human error and traffic accidents.

Autonomous driving technology [1,2] is currently one of the most popular research fields. It combines multiple advanced technologies and has a revolutionary impact on the entire automotive industry. Major automobile manufacturers worldwide are actively exploring autonomous driving technology such as Tesla’s Autopilot [3], an automatic assistance driving function. Autonomous driving systems contain many functions, among which the recognition of gestures used by police officers is important and an essential task for self-driving cars to reach the L4 level or higher. These levels were set by J3016 [4], which defined six levels of driving automation, ranging from no driving automation (level 0) to full driving automation (level 5). During road congestion, autonomous driving vehicles must be able to quickly and accurately recognize the hand gestures of traffic police, make appropriate decisions, and execute them to restore traffic flow and avoid further traffic congestion or accidents. Therefore, traffic-police gesture recognition technology has broad application prospects in modern traffic management, traffic safety, and autonomous driving fields. This technology can improve the long-term stability of traffic order, effectively respond to the challenge of rapidly increasing vehicle numbers on urban roads, and provide accurate guidance for autonomous driving vehicles, achieving intelligent traffic guidance and management. The application of traffic-police gesture recognition technology can improve the efficiency and accuracy of traffic management, reduce human error and the occurrence of traffic accidents, and bring higher safety and reliability to autonomous driving vehicles. The development of autonomous driving technology provides a broader application scenario for traffic-police gesture recognition technology, which can be applied to intelligent transportation systems and autonomous driving vehicles to realize more intelligent and efficient transportation.

Currently, there are three main methods for human motion recognition: wearable devices [5], computer vision (CV) [6], and wireless signals [7,8]. Wearable devices embed sensors, including accelerometers, gyroscopes, and wireless radio modules, into items worn daily such as wristbands and watches to extract data collected by the sensors to recognize various human motions and postures. Human posture recognition based on computer vision relies on cameras to capture video time sequences of human motions. The data are classified through image processing and feature extraction, enabling the recognition of different human motions. However, using wearable devices for data collection may cause inconvenience to users’ daily lives. Furthermore, wearable devices require a power supply, which can make them unable to work continuously for long periods. Additionally, this method has limitations in low-light environments, and when the camera is blocked, it fails to obtain data, making it unable to function correctly in dark environments. Moreover, this method poses certain privacy and security risks to users.

In contrast, as a solution, wireless signals can effectively overcome the inconvenience, unsustainable power supply, and high light requirements associated with wearable devices. Wireless signals collect echo data reflected from the target and protect human privacy while remaining unaffected by light, weather, etc. They can work for extended periods without frequent maintenance. Furthermore, as a non-invasive technology, wireless signals do not raise privacy concerns and are gaining attention in both industry and academia [9,10,11].

In order to address the aforementioned issues and challenges, this article presents mm-TPG, a traffic-police gesture recognition system that is based on millimeter-wave radar point clouds. The system, named mm-TPG, utilizes a 60 GHz frequency-modulated continuous-wave millimeter-wave radar as a sensor to accurately detect traffic-police gesture movements. Firstly, the system initially acquires information about human motion by synthesizing the intermediate frequency signal and effectively eliminating clutter and noise. Secondly, it transforms the information of human motions into point cloud data and subsequently determines the optimal number of frames for point cloud synthesis through rigorous testing. Finally, spatial features are extracted using the ResNet18 network, which is combined with gated recurrent units (GRUs), to further extract temporal features, thereby achieving accurate gesture classification. The main contributions of this article can be summarized as follows:

- A signal-noise removal method with double-threshold detection is proposed, which effectively solves the problem of noise and clutter in the data and achieves the enhancement of radar signal and echo information.
- A model that introduces multi-frame point cloud synthesis and combines ResNet18 with gated recurrent units, addresses the problem of the limited information capacity of single-frame point clouds, improves the efficiency of classification, and avoids the problem of gradient explosion.
- The mm-TPG system developed in this paper senses the command gestures of traffic police in a non-contact manner. Through extensive experiments, it is proved that the system is almost unaffected by personnel differences and has a comprehensive recognition rate of over 89% with strong robustness.

## 2. Related Works

Non-contact methods are widely used in human action recognition, including recognition based on RGB and Kinect cameras, as well as features extracted from wireless signals. Some related studies, such as those by Wachs et al. [12] and Rautaray et al. [13], have proposed a computer vision (CV)-based gesture perception approach. Xiong et al. [14] proposed a gesture skeleton extractor (GSE) to extract gesture features by recognizing RGB images, and a multi-channel extended graph convolutional network was used for gesture recognition. Guo et al. [15] also used an individual Kinect camera to obtain RGB images and depth information on human posture, constructed static descriptors based on depth data, and estimated dynamic descriptors based on an RGB image sequence. The author fused two descriptors to generate a new description feature that combines motion and stillness, using average structural similarity indices to recognize actions. Zhou et al. [16] proposed a real-time recognition method for hand poses in color-depth images and computed a histogram of hand-pose gradient features to enable an extreme learning machine classifier to recognize different poses. Although integrating visual sensors can provide more accurate information about human body part positions, thereby improving the accuracy of gesture recognition, this method is only applicable in well-lit environments. Its efficacy is significantly compromised in low-light or dark environments, and privacy issues cannot be avoided. Therefore, solutions must be developed to overcome these problems and challenges.

RF gesture recognition can collect wireless signals using devices such as RFID, WiFi, and radar. In [17], the authors proposed a deep learning-based human motion-sensing architecture using RFID. Abdelnasser et al. [18] proposed an indoor human-action recognition framework using WiFi signals that detects daily indoor activities by employing fusion algorithms based on the received signal strength indicator (RSSI). Additionally, Wang et al. [19] proposed E-eyes, which uses channel-state information (CSI) to recognize nine daily activities, such as bathing and sleeping. Nonetheless, due to the low frequencies and long wavelengths of WiFi and RFID signals, they cannot provide high-resolution gesture data. Compared to other gesture recognition methods, millimeter-wave radar sensing technology has several advantages, including:

- Millimeter-wave signals have higher frequencies and shorter wavelengths, providing higher spatial resolution and more detailed gesture data.
- Millimeter-wave point cloud data not only includes traditional features such as distance and angle but also directly indicates the spatial position of the target, making the gesture information more visualized.
- Millimeter-wave radar can obtain high-quality data even in harsh weather conditions, independent of weather and lighting conditions.

In recent years, millimeter-wave radar sensing technology has been widely used in fields such as human pose recognition, motion tracking, target identification, and intelligent driving [20,21,22,23]. For practical applications, extracting relevant data features from the reflection signals is key for millimeter-wave radar. Therefore, researchers have extensively explored data processing methods to extract more detailed features from radar echo signals, such as distance [24] and angle [25]. Some studies have used the Orthogonal Matching Pursuit (OMP) algorithm to analyze radar echo signals and obtain micro-Doppler trajectories and then employed neighborhood classifiers to classify gestures [26]. Other research utilized radar to detect the micro-Doppler information of targets and further applied convolutional neural networks (CNN) to detect and classify human motion behaviors [27]. Pavlo et al. [28] used the data received by the short-range FMCW radar system with the range-Doppler map estimation method and the depth-sensor calibration system to complete vehicle gesture recognition. Dekker et al. [29] processed the one-dimensional signal of hand gestures into the Doppler time spectrum, taking the real and imaginary parts of this spectrum as the input of the convolutional neural network, respectively, to complete the classification and recognition of hand gestures. Wang et al. [30] obtained their two-dimensional distribution in the range-Doppler domain by successively performing declination, fast time domain fast Fourier transform, and coherent integration processing on the radar echo of the gesture target. They used it as the input feature vector of the subsequent deep learning network to achieve automatic feature extraction and recognition of gesture actions. The above methods can achieve good recognition effects in simple scenes, but when there are many confounding factors, the key features in the spectrum may become blurred, making it difficult to distinguish. By using the mean phasor cancellation algorithm and improved CFAR algorithm, the method proposed in this paper can effectively filter out interference from static targets and extract dynamic targets with strong reflection intensity, making it more suitable for complex traffic scenes. Compared with other gesture recognition methods, the advantage of the method proposed in this paper is that the extracted point cloud data not only contains the most traditional features but can also directly display the spatial position of the target, making it more attractive. For example, mPoint uses FMCW signals and Multiple Input Multiple Output (MIMO) millimeter-wave radars to generate three-dimensional point cloud data of human targets with good generation results [31]. Additionally, a new non-intrusive human activity recognition system that converts millimeter-wave radar into point clouds can learn activities through dual-view convolutional neural networks and achieve high-accuracy fall detection classification results [32]. Researchers have also used the spectrum of millimeter-wave radar echoes as input and improved classification accuracy through an acceleration method based on a Field-Programmable Gate Array (FPGA)-accelerated CNN [33]. In the field of traffic-police gesture recognition, there are also related studies. For example, one study using a point cloud classification network employed traffic-police gestures as training data and achieved recognition of four gestures: parking, turning right, turning left, and holding [34]. In contrast, this paper uses millimeter-wave point clouds to study the recognition of six traffic-police gestures. Based on the above works, we developed the traffic-police gesture recognition system, which utilizes a 60GHz FMCW millimeter-wave radar as a sensor. The radar has high spatial resolution and operates independently of weather and lighting conditions. As a result, it can obtain high-quality point cloud data. At the same time, the mm-TPG system also adopts a series of effective data processing methods, such as static target denoising, feature extraction, and classification recognition. Experimental results show that the system can accurately recognize various traffic-police gesture movements with high accuracy and robustness, and has an essential role in practical traffic command.

## 3. System Design

### 3.1. Experiment Setup

**Figure 1:**
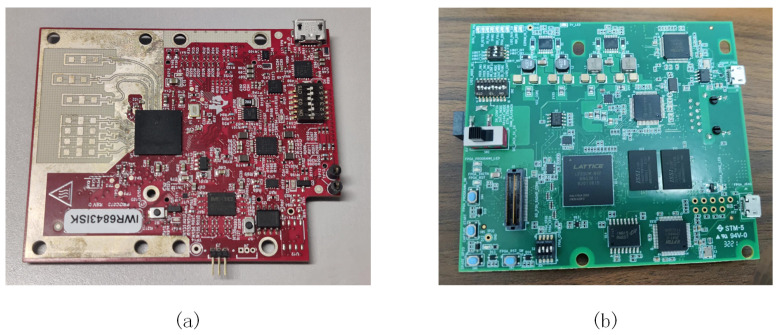
Experimental equipment: (**a**) IWR6843ISK. (**b**) DCA100EVM.

In this experiment, we used the IWR6843ISK millimeter-wave radar, as shown in Figure 1a, which is a single-chip millimeter-wave radar sensor with three transmit and four receive antennas, a built-in phase-locked loop (PLL) and an analog-to-digital converter (ADC). The radar device integrates TI’s high-performance C674x DSP for radar signal processing with a frequency range of 60 GHZ–64 GHZ. The maximum continuous bandwidth of the radar is 4GHz, which corresponds to a range resolution of 3.75 cm. The radar sets the radar transmit signal start frequency to 60 GHZ. The radar has three transmit antennas and four receive antennas, an ADC sampling rate of 2.95 × 10,696 chirps per frame, each frame with a duration of 55 ms, and is set to 1000 frames. The IWR6843ISK millimeter-wave radar is connected to the DCA1000EVM data acquisition board (as shown in Figure 1b) to obtain the raw ADC data and transmit the data to the computer via a USB data cable. When the computer receives the raw data, MATLAB is used to parse and process the data using hardware configured with an I7-8750H and a GTX1060.

### 3.2. Overview

**Figure 2:**
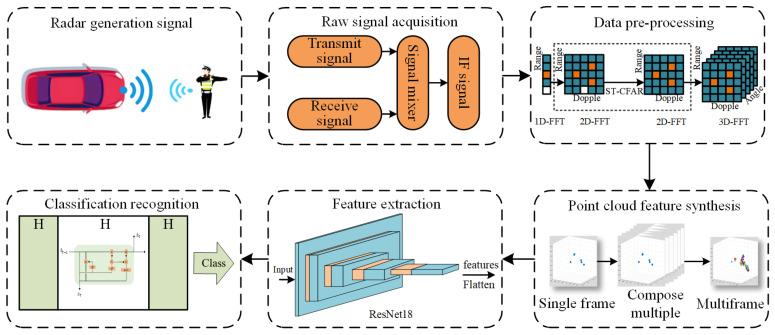
Overview of the mm-TPG system.

The structural diagram of the mm-TPG system is shown in Figure 2. This system utilizes millimeter-wave radar to emit continuous frequency-modulated waves and receive reflected signals. By analyzing these signals, point cloud data is generated, thereby achieving the task of recognizing traffic-police gestures. The main workflow of the system is as follows. First, the IWR6843ISK millimeter-wave radar board is used to collect the raw echo data of the front traffic-police gesture, and then the intermediate frequency signal is synthesized for the raw signal. Multiple Fourier transforms, static clutter filtering algorithms, and constant false alarm calculations are then used to obtain the position information of the target. The position information of the target is transformed into a Cartesian coordinate system, and the point cloud features of the gesture are generated using a multi-frame point cloud synthesis method. In the classification module, the system first uses the ResNet18 network to extract the spatial features of point cloud data. Then, gated recurrent units (GRUs) are used to extract the temporal features of the multi-frame point cloud data. Finally, the extracted features are classified using a classifier to achieve accurate recognition of traffic-police gestures.

### 3.3. Raw Data Pre-Processing Module

**Figure 3:**
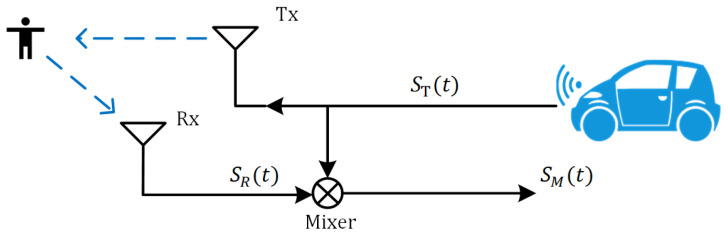
IF signal synthesis process.

FMCW radar is a widely used passive radar system that uses the principle of frequency modulation to acquire the target information. This radar system works by transmitting continuous frequency-modulated signals with broadband, low-power, and continuous-emission characteristics. Compared with other radar systems, FMCW radar signals have better anti-jamming performance and can achieve target detection and localization by processing the FM difference of the received echoes. In millimeter-wave radar, a linear FM signal is usually used as the transmit signal, which means that the frequency of the signal varies continuously over time and is modulated in an incremental or decremental manner. The linear FM signal can be expressed using Equation (1) [35]:

(1) $$s_{T}\left(t\right)=A_{T}\cos[2\pi f_{c}t+2\pi \frac{B}{T}\int_{0}^{t}\left(t-\tau \right)m_{T}\left(\tau \right)d\tau ]$$

where $A_{T}$ is the amplitude of the transmitted signal, $f_{c}$ is the center frequency of the signal, *B* is the FM bandwidth, *T* is the period of the transmitted signal, and $m_{T}\left(t\right)$ is the modulation function of the FM signal. Then, the transmitted FM signal is sent to the antenna for radiation, and after propagation through the air, it is reflected by the target object. The reflected signal is received by the antenna and can be expressed using Equation (2) [35]:

(2) $$s_{R}\left(t\right)=A_{R}\cos[2\pi f_{c}\left(t-\tau \right)+\varphi ]$$

where $A_{R}$ is the amplitude of the received signal, τ is the distance from the target object to the radar, and ϕ is the initial phase of the received signal. After the reflection from the target object, part of the signal is received by the receive antenna and fed into the mixer through the receiving signal channel. The mixing process is shown in Figure 3. The essence of the mixer is to multiply the transmitted signal and the received signal to obtain a product signal. The frequency of this product signal is determined by the frequency of the transmitted signal and the frequency of the received signal, which is $f_{IF}=f_{r}+f_{t}$, where $f_{IF}$ is the frequency of the IF signal, $f_{r}$ is the frequency of the received signal, and $f_{t}$ is the frequency of the transmitted signal. Subsequently, the mixing process of the transmitted and received signals is used to generate the IF signal.

Then, the transmitted signal is multiplied by the received signal to obtain a new signal containing the sum of the two signal frequencies, as shown in Equation (3) [35]:

(3) $$s_{M}\left(t\right)=\frac{A_{T}A_{R}}{2}[\cos(2\pi f_{c}\tau )+\cos(2\pi f_{c}t+\varphi )]+\frac{A_{T}A_{R}}{2}m_{T}\left(t\right)\cos[2\pi f_{c}\left(2\tau +t\right)+\varphi ]$$

Finally, the analog signal is converted into a digital signal using an analog-to-digital converter.

### 3.4. Motion Feature Extraction Module

**Figure 4:**
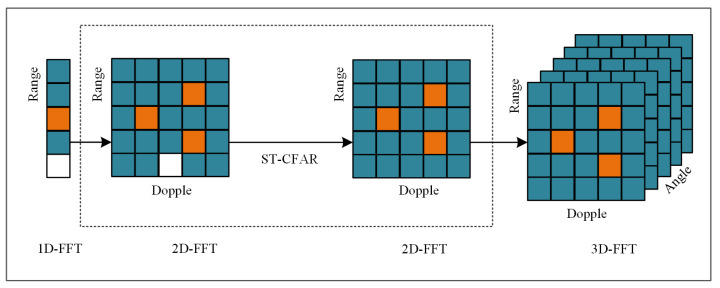
ST-CFAR schematic.

Millimeter-wave radar point clouds can be obtained by receiving echo signals and converting them for signal processing. In traffic-police gesture recognition, the millimeter-wave radar point cloud can be used to obtain the position information and movement status of the human body in space, such as the movement trajectory of the arm, the degree of arm extension and flexion, and the shape of the gesture, to realize the recognition of the traffic-police gesture. In order to extract the position information of the human body more accurately and efficiently, this paper proposes a method of signal-noise removal with double-threshold detection ST-CFAR, as shown in Figure 4. This method solves the problem of noise and clutter in the data and enhances radar signal and echo information.

**Figure 5:**
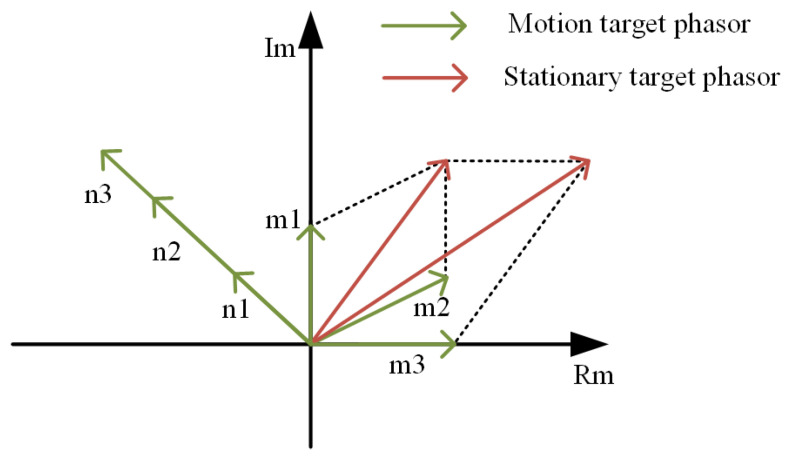
Mean phase elimination algorithm model.

Before synthesizing the point cloud operation, it is first pre-processed using the mean phase cancellation algorithm, as shown in Figure 5. In this algorithm, all the received pulses are averaged to derive the reference received pulse, and then the target echo signal can be obtained by subtracting the reference received pulse from each received pulse. The reference received pulse can be expressed using Equation (4):

(4) $$R\left[i\right]=\frac{1}{N}\sum_{j=1}^{N}R\left[i,j\right]$$

If the target is stationary, the phase of the chirp echo signal is the same, and the average value obtained after summing up the phases will be very large, such as (n1+n2+n3)/3. Consequently, the signal amplitude of each chirp signal becomes very small after subtracting the average value. Assuming that the target is a moving target or a micro-movement target, the phase of each FM pulse signal is different due to the movement of the target. As a result, its phase summation will cancel out after summation, and the mean value will be very small, such as (m1+m2+m3)/3. Consequently, the amplitude of each chirp signal will not be affected much after the mean value is subtracted.

**Figure 6:**
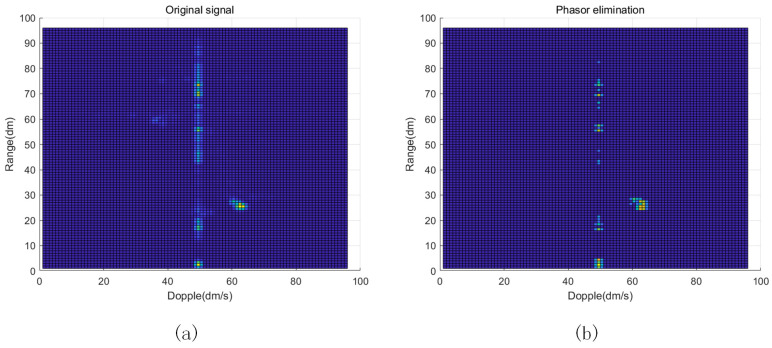
Mean phase elimination algorithm model. (**a**) Original signal. (**b**) After phase elimination.

The biggest difference between the mean phase cancellation algorithm and the commonly used MTI (Moving Target Indication) algorithm is the different ways they deal with static clutter. The MTI algorithm directly uses the phase difference to eliminate static clutter, whereas the mean phase cancellation algorithm averages and performs the difference operation to remove static clutter by superimposing the phase quantities. Therefore, the MTI algorithm only suppresses the phase difference of static targets and is less effective for moving targets. The results after the phasor cancellation suppression of the static targets are shown in Figure 6.

In contrast, the phase-volume mean phase cancellation algorithm greatly improves the signal-to-noise ratio of dynamic targets while suppressing the phase of static targets by finding the mean value. Then, in the next processing step, the cell-averaged constant false alarm rate (CA-CFAR) detection algorithm is used. This algorithm selects a local background window in the signal processing area by calculating the difference in distance between the target and the radar, which is used to estimate the statistical properties of the background noise. Based on the noise samples in the background window, a threshold value is calculated, which is usually based on statistical theory and is obtained by setting the false alarm probability and the background noise level. Then, the threshold value is set according to the statistical properties of the background noise of the signal. In this paper, the constant multiplication threshold method is used, i.e., the threshold value is set as the mean value of the signal in the reference window plus a constant multiplication factor (e.g., 3 times) multiplied by the standard deviation of the signal. By comparing the received signal with the pre-set threshold value, the presence or absence of the target signal can be determined. With these steps, the sensitivity of target detection can be improved as much as possible while keeping the false alarm probability constant. In short, these processing steps minimize the effect of noisy signals and allow for more accurate detection of target signals. With these algorithms and processing steps, the location information and action features of the human body can be effectively extracted to provide an accurate database for subsequent traffic-police hand-gesture recognition. The final metric for radar estimation is the angle of arrival (AoA), which is defined as the angle between the reflected signal and the horizontal plane. The angle estimation requires at least two RX antennas and is calculated as

(5) $$\theta_{r}=\frac{\lambda }{N_{r}\times N_{t}}d\cos\theta$$

where λ is the wavelength, $N_{r}$ is the number of receiving antennas, $N_{t}$ is the number of transmitting antennas, *d* is the distance, and cos θ is the cosine of the angle between the two receivers. The radar chip estimates the angle of arrival by using the phase change of the 2D FFT peak caused by the different distances of the object to each antenna. This FFT is called the Angle FFT, and it outputs the azimuth angle divided by the elevation angle. Finally, the radar receives multiple RX signals from all chirps sent by the TX antenna. After the above series of operations, the distance, azimuth, pitch, and velocity information of the target object can be obtained and set as (S,θ,ϕ,v), respectively. Then, the output under the spherical coordinate system is converted to the coordinates of the Cartesian coordinate system using Equation (6):

(6) $$\begin{array}{c} x=S\times \sin\varphi \times \cos\theta \\ y=S\times \sin\varphi \times \sin\theta \\ z=S\times \cos\varphi \end{array}$$

**Figure 7:**
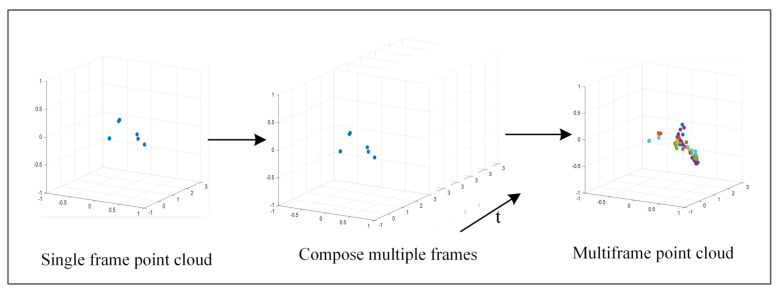
Multi-frame point cloud synthesis.

Due to the sparse nature of the millimeter-wave radar point cloud, the number of radar point clouds in a frame is small, which is not conducive to system recognition. Although the body movements are not reflected in a single frame, the arm movements build a spatio-temporal structure in the direction of motion in consecutive frames. These unique spatio-temporal structures in the point clouds vary from gesture to gesture and can be utilized for motion gesture recognition, as shown in Figure 7.

Then, we transform the 3D point cloud data into 2D image data, further compress the image to reduce the loss, and finally obtain the features with spatio-temporal characteristics. To reduce the computation, we map the 3D point cloud as 2D coordinates. Then, the mm-TPG feeds the generated point cloud action images into the gesture classification module to further classify them.

### 3.5. Classification Module

**Figure 8:**
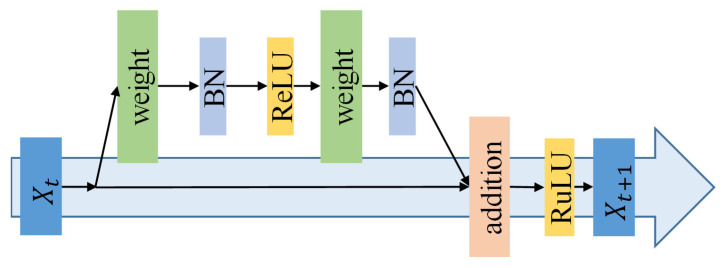
Residual block structure.

ResNet is a classical convolutional neural network structure, which is widely used in image classification tasks. It takes residual learning as the core idea and solves the gradient disappearance problem in deep networks using skip connections, with fewer parameters and a faster training speed. The structure of the residual blocks is shown in Figure 8.

**Figure 9:**
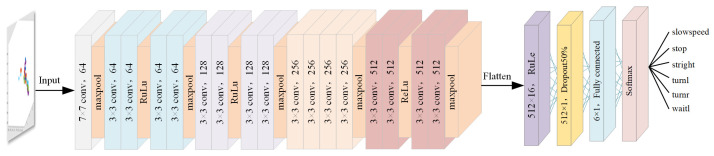
Overview of ResNet18 and GRU.

The structure of the ResNet18 used in this paper is shown in Figure 9. The model contains 18 convolutional layers, 16 of which are residual learning units. All convolutional operations are performed using a 3 × 3 convolutional kernel. The input is an image containing point cloud information. After each convolutional layer and before the activation function, batch normalization is used to accelerate convergence and avoid the problem of gradient disappearance or gradient explosion. The last two layers in ResNet18, namely the fully connected layer and the softmax layer, are deleted because, in the traffic-police gesture recognition task, we only need to extract features rather than directly classify them. The size of the output feature map for each frame is 1 × 1 × 512, and then it is spread out. These features are used as the input to the gated recurrent unit. By using ResNet18 for feature extraction, we can extract useful spatial information from the point cloud. ResNet18 has fewer parameters and a faster training speed for the traffic-police gesture recognition task. The extracted features are used for subsequent temporal feature extraction and gesture classification. By performing shaping and spreading operations on the output of the last residual block, we can obtain the feature vector y = [y1, y2, …, yT], and the total number of feature vectors is T = 512.

**Figure 10:**
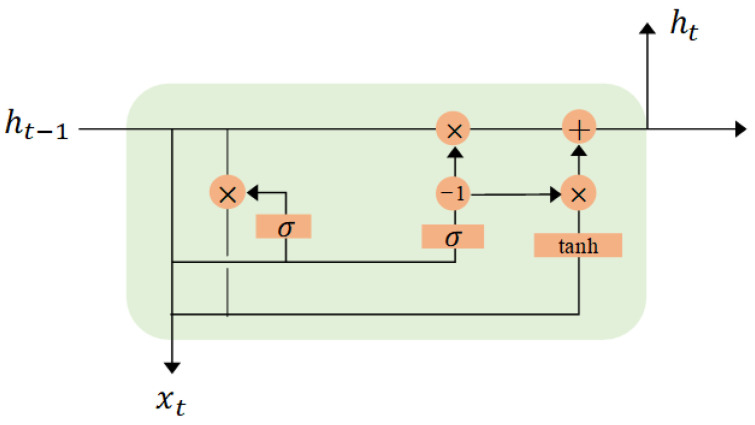
GRU network structure diagram.

In sequence modeling tasks, the traditional Recurrent Neural Network (RNN) faces problems such as gradient disappearance and gradient explosion. To solve these problems, we use Long Short-Term Memory (LSTM), which is a special type of RNN that uses “forgetting gates”, “input gates”, and “output gates” as controllers to control and retain information in the network and effectively solve the gradient disappearance and long-term dependency problems. Also, LSTM can better capture the contextual information and temporal features in the sequence. Then, to further solve the computational problems and model complexity in LSTM, we use GRUs. The GRUs adopt the memory unit mechanism used in LSTM, which only has two gates (reset gate and update gate), so it has fewer parameters and a faster computation speed compared to LSTM. The network structure of a GRU is shown in Figure 10.

In order to perform spatial feature extraction of human body parts, this paper proposes a model combining ResNet18 and doorway cyclic units to extract features. The ResNet18 model first splits each point cloud image into multiple small blocks, and then each of these small blocks is passed through the pre-trained model for feature extraction to obtain a feature vector of size 1 × 1 × 512. To further extract the time series features accurately and quickly, mm-TPG uses the GRU model to arrange these feature vectors in temporal order, forming a two-dimensional tensor. Next, this tensor is fed into the gating unit of the GRU model as an input for processing. To improve the generalization ability of the model, the dropout of the model is set to 0.5, and an Adam optimizer with a learning rate of 0.001 is used to train the deep learning classifier. Dynamic gesture features every 15 frames are used as input to the GRU. In the GRU model, the feature vector of each time step and the hidden state of the previous time step are passed to the gating unit, and the new hidden state and output of the current time step are calculated. Specifically, for each time step, the gating unit calculates a reset gate r and an update gate z based on the input and the hidden state of the previous time step. The reset gate determines how much of the input of the current time step and the hidden state of the previous time step should be retained, which is calculated using Equation (7) [36]:

(7) $$r_{t}=\sigma \left(W_{r}\cdot x_{t}+U_{r}\cdot h_{t-1}+b_{r}\right)$$

where $x_{t}$ denotes the input of the current time step; $h_{\left(t-1\right)}$ denotes the hidden state of the previous time step; and $W_{r}$, $U_{r}$, and $b_{r}$ are the learnable weights and biases in the gating unit, respectively, denoting the Sigmoid function. The update gate, on the other hand, determines how the input of the current time step should be integrated into the hidden state and is calculated using Equation (8) [36]:

(8) $$z_{t}=\sigma \left(W_{z}\cdot x_{t}+U_{z}\cdot h_{t-1}+b_{z}\right)$$

Next, the candidate hidden state $h_{t}^{\prime }$, which uses another set of weights and biases to weight the input and the hidden state of the previous time step, is calculated using Equation (9) [36]:

(9) $$h_{t}^{\prime }=\tanh\left(W\cdot x_{t}+r_{t}\cdot \left(U\cdot h_{t-1}\right)+b\right)$$

Then, the new hidden state $h_{t}$ and the output $y_{t}$ of the current time step are obtained by linearly combining the candidate hidden state *h* of the current time step and the hidden state $h_{t-1}$ of the previous time step by updating gate z, respectively, using Equation (10) [36]:

(10) $$h_{t}=\left(1-z_{t}\right)\cdot h_{t-1}+z_{t}\cdot h_{t}^{\prime }$$

The final output, $y_{t}$, is calculated using Equation (11):

(11) $$y_{t}=h_{t}$$

where $y_{t}$ is the output of the current time step. The process of each time step in the entire GRU model is similar, and the parameters of the model are updated through gradient descent based on the error value calculated using the loss function during training with the backpropagation algorithm. During testing, test image sequence data are input, and the output of each time step is calculated sequentially in the GRU model. Since the classifier eventually needs to recognize 6 gestures, we design a fully connected neural network with 6 output neurons. Finally, the softmax operation is performed on the output of the fully connected layer to accurately classify and recognize different gestures.

#### Point Cloud Gesture Classification Module Algorithm

The pseudo-code of Algorithm 1 is as follows, which describes the algorithm flow of the point cloud gesture classification module in the mm-TPG system. First, the original point cloud information is extracted, and then the information provided by the point cloud is increased through a multi-frame synthesis of the point cloud. Next, the synthesized point cloud is sent to the ResNet18 network to extract the spatial information features of the point cloud, and then temporal features are extracted using gated recurrent units. Finally, the classification and recognition of traffic-police gestures are complete.
**Algorithm 1:** Point cloud gesture classification
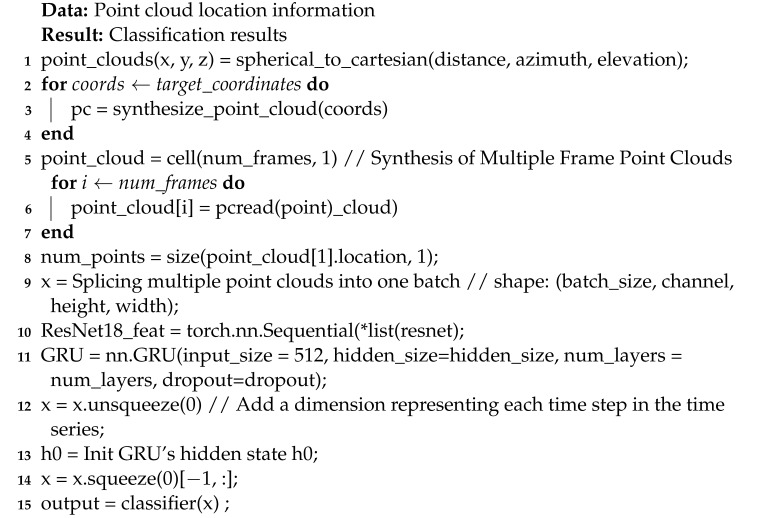


## 4. Experiments and Evaluation

In this section, an experimental evaluation of the mm-TPG system is performed to evaluate its overall performance by looking at different identification dimensions.

### 4.1. Experimental Analysis

#### 4.1.1. Effect of Different Distances on Experimental Results

**Figure 11:**
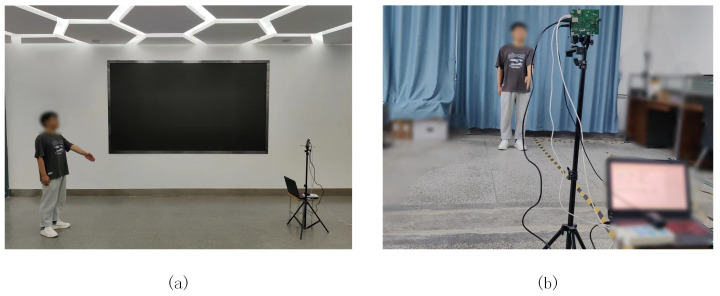
Real pictures of different environments. (**a**) Empty hall. (**b**) Laboratory.

A series of experiments were conducted to test the effect of human gestures on system performance in the spatial dimension. We selected six experimenters with heights of 1.5–1.88 m, with a male-to-female ratio of 1:1, to collect the radar echo data by performing standard traffic-police gestures. Each experimenter stood at a designated position and performed each of the six types of gestures gesture within 4 s, including stop, straight ahead, left turn, sit and wait for turn, right turn, and slow down, and a total of 3000 sets of samples were collected at different orientation angles and distances, with a sample ratio of 8:2 between the training and test sets. During the experiment, the subjects were placed in two environments, a laboratory and an empty hall. The real environment of the experimental scene is shown in Figure 11, in which Figure 11a is the laboratory environment and Figure 11b is the empty hall environment.

**Figure 12:**
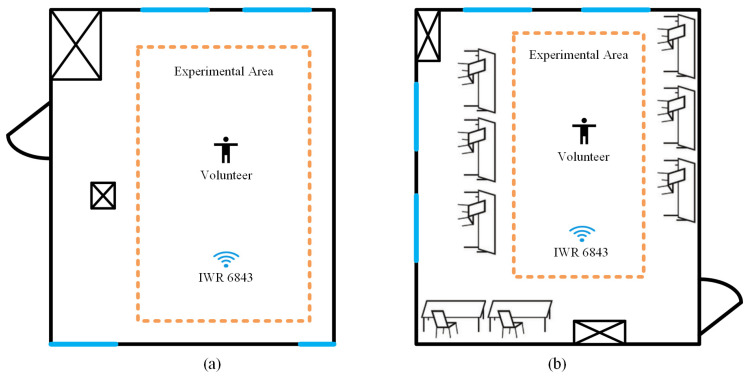
Different environments. (**a**) Empty hall. (**b**) Laboratory.

The schematic diagram of the experimental environment is shown in Figure 12, Figure 12a shows a relatively simple empty hall environment and Figure 12b shows a relatively complex laboratory environment.

**Figure 13:**
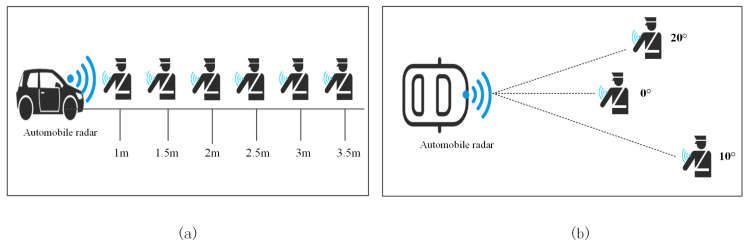
Schematic diagram of the spatial dimension experiment. (**a**) Different distances. (**b**) Different arrival angles.

We considered different distances and angles of arrival. Figure 13a shows the experimental schematic for testing the performance of the gesture recognition method at different distances. In this experiment, we placed subjects at different distances to evaluate the system’s ability to recognize gestures for near and far targets. By adjusting the distances, we can observe the performance of the system at different distances and analyze the effect of distance on the accuracy of gesture recognition. Figure 13b shows the experimental schematic for testing the performance of the gesture recognition method at different arrival angles. In this experiment, we adjusted the angle of human gestures reaching the radar to simulate different gesture action situations. By varying the arrival angle, we can evaluate the system’s ability to recognize gestures varying in spatial dimensions and further explore the effect of gesture orientation on system performance.

a Different distances

**Figure 14:**
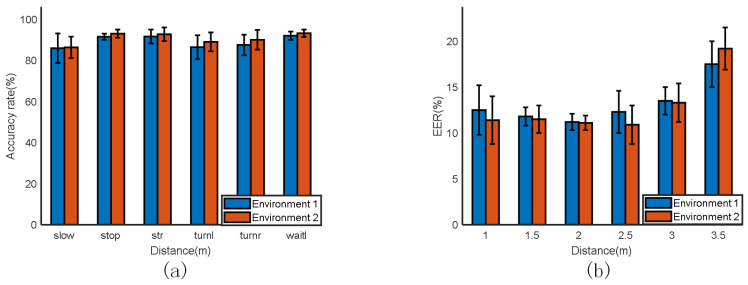
(**a**) The effect of different environments on the recognition rate. (**b**) The effect of different distances on the recognition rate.

We first asked the experimenter to make six traffic-police gestures in two environments. The experimental results are shown in Figure 14. We set the device distance at 1 m, 1.5 m, 2 m, 2.5 m, 3 m, and 3.5 m to test the performance of mm-TPG in order to find the best device distance. The results are shown in Figure 14b. As shown in Figure 14a, the accuracy rate achieved in the empty hall was higher than that in the laboratory, which was more complex and had additional interference. According to the final results, the average gesture recognition rate was higher than 87% in both environments, indicating that mm-TPG has good recognition performance for gestures. As can be seen in Figure 14b, the recognition rate in the empty hall was always higher than that in the laboratory, no matter how far apart the devices were. The recognition rate was the highest when the radar was about 2 m away from the subject, both in the empty hall and in the laboratory. The signal propagation distance was short, the signal attenuation was small, and the sensing range was wide. When the device distance was equal to or greater than 3.5 m, as the distance increased, the number of point clouds reflected by the human body decreased, resulting in a decrease in the information volume and difficulty in extracting effective features, thereby affecting the accuracy of action recognition. Similarly, as the distance increased, the minimum distance between two targets that the system could distinguish also increased, which affected the resolution of the radar sensor and reduced the accuracy of gesture recognition.

b Different azimuth angles

**Figure 15:**
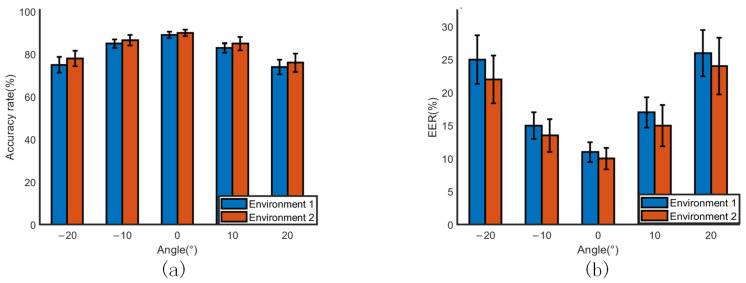
Effect of different angles on recognition rate. (**a**) Accuracy rate. (**b**) Error rate.

Since in actual scenarios, traffic policemen sometimes face the car at a certain angle when performing hand-gesture commands, we set different arrival angles of 0°, ±10°, and ±20° to test the performance of mm-TPG, and the experimental results are shown in Figure 15. The results demonstrate the effect of the environment and angle of arrival on the gesture recognition rate. As can be seen in Figure 15a, the overall recognition accuracy rate reached 90% when the experimenter made gestures with an azimuth angle of 0° to the device, and the error rate of gesture recognition was only 10%. When the azimuth angle between the experimenter and the device was ±10°, the error rate of gesture recognition did not differ much and fluctuated around 15%. When the azimuth angle between the experimenter and the device was ±20°, the performance of gesture recognition decreased more obviously, and its error rate fluctuated around 30%. This is because the radar can sense more echo information if the movement amplitude is in the radial direction of the radar when the gesture action is performed. Overall, mm-TPG was able to achieve good recognition performance while accommodating smaller directional angle errors.

#### 4.1.2. Influence of Different Subjects

**Figure 16:**
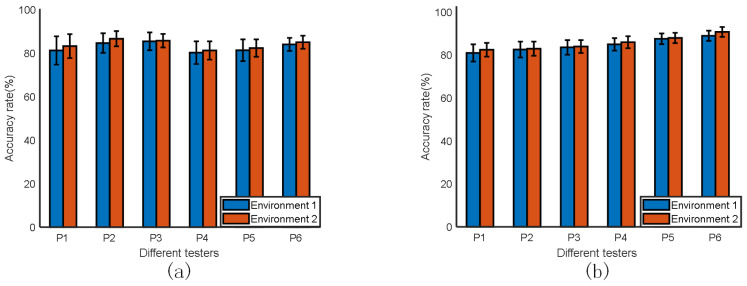
Effect of different subjects. (**a**) Test results for untrained personnel. (**b**) Test results for personnel of different heights.

To investigate the effect of different subjects on the recognition rate more thoroughly, we randomly selected one subject from the original group of experimenters and removed them. We then collected data from and trained the remaining five subjects in the same experimental scenario. Next, we used the transferred subject’s data to conduct a test, and the results are shown in Figure 16a. After that, we invited six new subjects to participate in the experiment, and we sorted them according to their height, from lowest to highest. We collected and validated the data from these six subjects as a test set. As seen in Figure 16a, the gesture samples from the untrained samples also achieved good recognition results compared to the trained samples. The average recognition rate for the untrained samples was 83%. From Figure 16b, it can be seen that in the test data of the same untrained subjects, the experimental results were proportional to the increase in height. This is because, in general, taller people have longer arm movements compared to shorter people. Consequently, in the radar radiation range, the arm movements of taller people are more likely to be reflected, generating more point clouds, which is more favorable for the extraction of point cloud features. Therefore, the recognition rate is expected to increase to some extent. Overall, this set of comparison experiments shows that the mm-TPG system has less influence on the individual variability of the experiment, and the subsequent application of the system can effectively reduce the data collection workload for future users.

#### 4.1.3. Effect of Different Numbers of Synthesized Frames on the Experiment

Although the movement characteristics of the body are not obvious in a single frame, the motion of the arm builds a spatio-temporal structure in the direction of consecutive frames, so we evaluated the performance of mm-TPG by synthesizing different numbers of consecutive frames. In Table 1, it can be seen that the experimental results were very obvious for the different numbers of frames synthesized together. The data synthesized in 1–3 frames, although the processing time was relatively short, exhibited relatively low recognition accuracy. However, as the number of frames synthesized increased, the overall recognition rate rose to above 80% when four frames were synthesized. With a further increase in the number of frames synthesized, the recognition rate gradually increased, reaching the value of 89.6% when six frames were synthesized, after which the accuracy started to decline. This is because too many frames of point cloud synthesis will cause a lot of information redundancy, resulting in a decrease in recognition accuracy. Additionally, with the increase in the number of synthesis frames, the pre-processing time also increases, which is obviously not suitable for our system. Therefore, it can be concluded that the highest recognition rate of mm-TPG is achieved when the number of synthesized frames is six, and the data pre-processing time is within an acceptable range.

#### 4.1.4. Comparison of Different Feature Extraction Models

**Figure 17:**
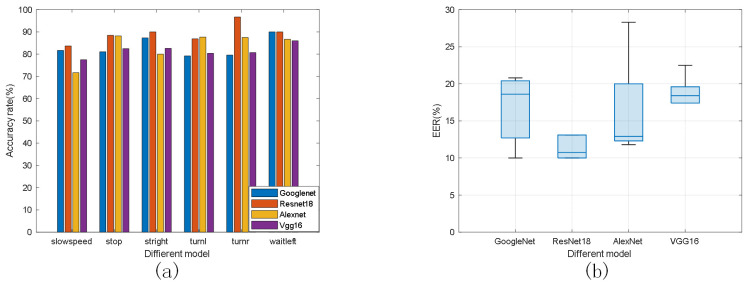
Comparison of different models. (**a**) Recognition rate of each gesture. (**b**) Error rate.

In order to test the performance of different feature extraction models, we evaluated the effects of different spatial feature extraction models on the experimental results. The sample data of gestures were used in the experiments, and the ratio of training samples to test samples was 4:1. The comparison results are shown in Figure 17. In Figure 17a, we can see that AlexNet achieved high accuracy in feature extraction for stopping and going straight, but low accuracy in feature extraction for deceleration. VGG16 achieved low accuracy in feature extraction overall. GoogleNet achieved an accuracy rate of higher than 83% in feature extraction. The ResNet18 network used in this paper achieved an accuracy rate of only 86% in feature extraction for deceleration, but the accuracy rate in feature extraction for other actions was above 90% and the overall recognition rate reached 89%. Figure 17b shows the EER of the four models, and the results demonstrate that the effect of ResNet feature extraction is better compared to the other models.

#### 4.1.5. Comparison with Other Existing Methods

To verify the performance of the mm-TPG method, we compared our results to those of the Zhang et al. [31], Wi-Num [37], and MMPointGNN [34] methods in an empty hall environment, respectively. Zhang et al’s method employs a convolutional neural network for the detection and classification of human motion behavior using micro-Doppler information collected through radar. The WiNum method uses discrete wavelet transform for CSI data noise reduction, the AGS algorithm for the adaptive segmentation of gestures, and the gradient boosting decision tree (GBDT) integrated learning algorithm for gesture recognition. MMPointGNN employs the PointNet network for the recognition of four gestures of traffic policemen (stop, right turn, left turn, and hold) by pre-processing the raw data and matching the reflected points of human bones with the target. As shown in Table 2, the WiNum method achieved the worst performance, as it ignored the CSI information features and utilized GBDT for classification by directly feeding the segmented data to the GBDT. Zhang et al.’s method achieved a slightly higher recognition rate compared to the WiNum method and classified using the micro-Doppler features of gesture movements. However, it was susceptible to interference and required better environmental conditions. MMPointGNN achieved a higher recognition rate for four specific gestures, but the combined recognition rate for all six types of traffic-police gestures was low, at only 84%. However, our method extracted key features of traffic-police gestures in space and time, and the filtering algorithm in the pre-processing stage removed most of the environmental noise. Therefore the final recognition rate was better compared to the other methods.

## 5. Conclusions

In this paper, a traffic-police gesture recognition method, known as mm-TPG, which is based on a millimeter-wave radar point cloud, is proposed. The method directly utilizes point cloud data collected by automotive radar, eliminating the need for additional sensors and reducing cost and complexity. At the same time, the method can be adapted to various weather and lighting conditions to ensure the stability and reliability of the data. After a large number of verification experiments, mm-TPG achieved an accuracy rate of more than 89% in recognizing traffic-police gestures, which proves the feasibility of using a millimeter-wave radar point cloud for recognizing traffic-police gestures. This paper provides a novel idea and method for the intelligent autonomous driving field, which is expected to play an important role in practical applications. However, real traffic environments are very complex and ever-changing, and the data types and scenarios documented in this article are relatively low in complexity. Subsequent studies are needed to increase the complexities of the scenes, such as adding static targets and obstructions ahead and placing the radar in the car as in a real environment to conduct further experiments. This will enable us to adapt to a more realistic and complex traffic environment in the future. In addition, although millimeter-wave radar has many advantages, it also has the disadvantage of low resolution compared to cameras. Therefore, in our future research, we will combine millimeter-wave radars and cameras to obtain information, so that the advantages and disadvantages of the two can complement each other.

## Figures and Tables

**Table 1:** Effect of different numbers of synthesized frames on experimental results.

Number of Frames	Preconditioning Time	Accuracy
1	0.029	52.90%
2	0.042178	66.1%
3	0.058164	74.60%
4	0.07059	80.3%
5	0.086115	88.2%
6	0.101581	89.60%
7	0.125181	87.10%
8	0.135551	84.50%
9	0.156078	78.60%

**Table 2:** Comparison of recognition accuracy of each model.

Project	Device	Algorithm	Feature	Accuracy
Zhang et al.	mmWave	CNN	micro-Doppler	81%
Wi-Num	WiFi	GBDT	CSI	80%
MMPointGNN	mmWave	PointNet	Pointcloud	84%
mm-TPG (ours)	mmWave	ResNet+GRU	Pointcloud	89%

## Data Availability

Not applicable.

## Abbreviations

The following abbreviations are used in this manuscript:

FMCW	Frequency-Modulated Continuous Wave
SAE	Society of Automotive Engineers
GRU	Gated Recurrent Units
CV	Computer Vision
GSE	Gesture Skeleton Extractor
RFID	Radio Frequency Identification
RSSI	Received Signal-Strength Indicator
CSI	Channel State Information
OMP	Orthogonal Matching Pursuit
CNN	Convolutional Neural Networks
MIMO	Multiple Input Multiple Output
FPGA	Field-Programmable Gate Array
MTI	Moving Target Indication
AoA	Angle of Arrival
FFT	Fast Fourier Transform
